# A meta-analysis of vitrectomy with or without internal limiting membrane peeling for macular hole retinal detachment in the highly myopic eyes

**DOI:** 10.1186/s12886-016-0266-5

**Published:** 2016-06-13

**Authors:** Xinxiao Gao, Jia Guo, Xin Meng, Jun Wang, Xiaoyan Peng, Yasushi Ikuno

**Affiliations:** Department of Ophthalmology, Beijing Anzhen Hospital, Capital Medical University, Beijing, China; Beijing Institute of Ophthalmology, Beijing Ophthalmology and Visual Science Key Lab, Beijing Tongren Eye Center, Beijing Tongren Hospital, Capital Medical University, Beijing, China; Department of Ophthalmology, Osaka University Graduate School of Medicine, Osaka, Japan

## Abstract

**Background:**

To evaluate the anatomical and visual outcomes by par plana vitrectomy with or without internal limiting membrane (ILM) peeling in highly myopic eyes with macular hole retinal detachment (MHRD).

**Methods:**

MEDLINE (Ovid, PubMed) and EMBASE were used for data collection up to September 30, 2015. The parameters of anatomical success, macular hole closure and improved best corrected visual acuity (BCVA) at or beyond 6 months after operation were assessed as the primary outcome measurement. The meta-analysis was performed with the fixed-effects model.

**Results:**

Seven comparative analyses involving a total of 373 patients were included in the present meta-analysis. Statistically the pooled data showed significant relative risk (RR) in terms of primary reattachment between ILM peeling and non-peeling groups (RR, 1.19; 95 % CI, 1.04 to 1.36; *P* = 0.012). An effect favoring ILM peeling with regard to macular hole closure was also detected (RR, 1.71; 95 % CI, 1.20 to 2.43; *P* = 0.003). However, no statistically significant difference was found in the improved BCVA (logarithm of the minimum angle of resolution) at 6 months or more (95 % CI, −0.31 to 0.44; *P* = 0.738).

**Conclusions:**

There is no proved benefit of postoperative visual improvement. However, the available evidences from this study suggested a superiority of ILM peeling over no peeling for myopic patients with MHRD.

## Background

Retinal detachment resulting from macular hole (MHRD) is one of the most vision-threatening complications to high myopias, with the incidence reported to be 67.7 % in myopic eyes between −8D and −3.25D [1]. Although the pathogenesis is not completely understood, the elongation of the axial length, the posterior staphyloma formation, the tangential traction of vitrous cortex and epiretinal membrane may be related factors [2, 3]. The rigidity of the internal limiting membrane (ILM) has also been considered to be an important factor [4]. For this reason, par plana vitrectomy (PPV) combined with ILM peeling was believed to be one of the beneficial procedures for those patients. The rationale includes relieving macular traction completely and increasing the flexibility of retina to conform better to the posterior staphyloma [5].

There were growing studies supporting the benefit of ILM peeling for myopic MHRD [6, 7]. However, the conflict results also found from other authors there were no significant association between ILM peeling and primary anatomical reattachment or macular hole closure rate [8–10]. In addition, ILM removal has been shown technically more challenging in patients with myopic maculopathy, even assisted with perfluorocarbon liquids for flattened retina and with the aid of vital dyes [11].

Evidences for comparing the benefits and complications of ILM peeling versus no peeling are largely lacking. Thus, in this study we compared anatomical and functional success rates of PPV with or without ILM peeling for MHRD in high myopias. Despite of the limitation of differences in the original study design, selection criteria and allocation protocol, in this meta-analysis we intend to design and make clear the optimal techniques by pooling available published studies.

## Methods

This meta-analysis was performed according to the Preferred Reporting Items for Systematic Reviews and Meta-Analyses (PRISMA) guidelines [12].

### Strategy for data collection

The databases of MEDLINE (Ovid, PubMed) and EMBASE were used for data collection up to September 30, 2015. The articles collected from these databases were related to MHRD in highly myopic eyes. The following search strategy was used: (“macular hole” OR MH) AND (“retinal detachment” OR RD) AND (myopi* OR myopia [mesh]) AND (vitrectomy) AND (“inner limiting membrane” OR “internal limiting membrane” OR ILM) AND (removal OR peeling). All studies were limited to the English language.

### Inclusion and exclusion criteria

All cases recruited for this study were considered to follow the included and excluded criteria. The inclusive criteria included a) studies that compared outcomes of par plana vitrectomy with ILM peeling vs. non-peeling in highly myopic eyes with MHRD; b) detailed description of surgical procedures; and c) the follow up period no less than 6 months. The exclusive criteria included a) previous history of PPV; b) presence of peripheral retinal break or proliferative vitreo-retinopathy before the primary surgery; c) use of silicone oil tamponade in the primary surgery; d) use of scleral buckling, including macular buckling in the primary study; and e) studies with inadequate records of postoperative reattachment rates, macular hole closure rates or postoperative best corrected visual acuity (BCVA) at ≥6 months.

### Data extraction

Two reviewers independently extracted the data that met the inclusion criteria. After the first extraction, data were rechecked and any disagreement regarding eligibility during the extraction was resolved by discussion. The information extracted from each study included the first author, publication year, country, trial type, age, gender, number of subjects, follow-up time, preoperative and postoperative BCVA. Intraoperative and postoperative complications were also recorded. The outcomes of interest that were extracted included the anatomical success, macular hole closure, and improved BCVA at ≥6 months.

### Outcome measurements

The measurements include a) Anatomical success was defined as the complete disappearance of the subretinal fluid and neurosensory retinal reattachment to the underlying retinal pigment epithelium; b) Macular hole closure was defined by optical coherence tomography as the complete disappearance of the hole and absence of neurosensory defect over the fovea; and c) The logarithm of the minimum angle of resolution (logMAR) improved BCVA at ≥6 months.

### Assessment of study quality

The quality of the included studies was assessed by two independent authors (XXG and JG) using a previously reported system by Downs and Blacks [13]. The system comprises 27 items distributed among 5 subscales regarding reporting (10 items), external validity (3 items), bias (7 items), confounding (6 items), and power (1 item). Discrepancies in the qualitative assessment were resolved by discussion until consensus was reached. The total score of each trial was expressed as a percentage of the maximum achievable score. Studies with a quality score of ≥50 % were considered to have adequate quality.

### Statistical analysis

The meta-analysis was conducted using the Stata software package (version 12.0; Stata Corp). The relative risk (RR) was measured with 95 % confidence interval (CI) for dichotomous variables, whereas the weighted mean difference (WMD) was calculated with the 95 % CI for continuous variables. A P value less than 0.05 was considered statistically significant. Statistical heterogeneity among trials was analyzed using the Pearson’s chi-square test and *I*^*2*^ tests. If there was significant heterogeneity between studies, a random-effects model was employed; otherwise, a fixed effects model was used to obtain summary RR or WMD.

## Results

### Data assessment

**Fig. 1:**
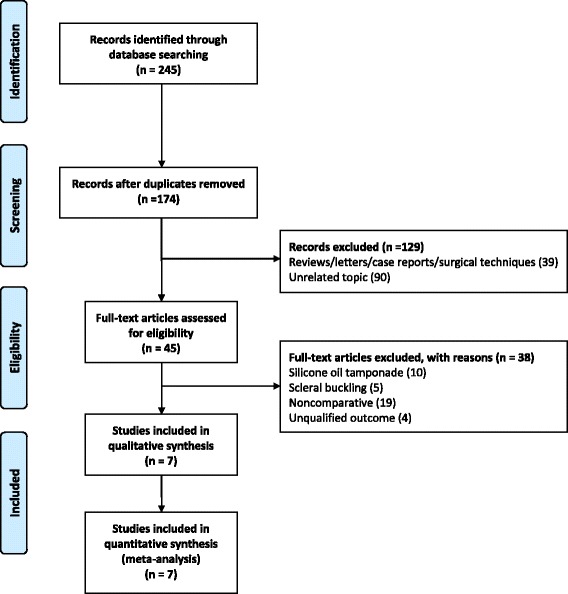
A flow diagram of strategies for the data collection

The search identified a total of 174 publications after duplicates removed. 129 studies were excluded after abstract evaluation. Of 45 publications that initially were considered potentially relevant, 38 finally were excluded. Seven comparative studies were included in the present meta-analysis involving a total of 373 patients who underwent PPV in highly myopic eyes with MHRD (Fig. 1). All studies fulfilled the quality criteria, with Downs and Blacks scores ≥50 % (Table 1).

**Table 1:** Characteristics and quality scoring components of included studies

Trial, year	Study design	Location	Number of eyes	Quality score component				Overall effect		
				I	II	III	IV	V	Overall	Percentage
Ikuno (2003) [14]	Retrospective, nonrandomized, comparative study	Japan	14/2	8	2	3	3	0	16	50.00 %
Lam (2006) [15]	Retrospective, interventional, comparative case series	China	44/13	9	2	2	3	2	18	56.25 %
Li KK (2010) [17]	Prospective interventional, comparative, case control study	China	10/9	10	2	3	2	0	17	53.13 %
Li X (2009) [9]	Prospective, randomized controlled, multicenter study	China	65/46	10	2	4	2	3	21	65.63 %
Nakanishi (2008) [10]	Retrospective, multicenter, interventional case series	Japan	40/9	9	2	2	2	2	17	53.13 %
Uemoto (2004) [18]	Retrospective, nonrandomized, comparative study	Japan	13/12	10	2	3	3	0	18	56.25 %
Wei (2013) [16]	Retrospective, comparative, case control study	China	47/49	10	2	3	3	2	20	62.50 %

### Surgical techniques

The surgical procedures were generally consistent with little difference in each study. Briefly, after the vitreous cortex was completely removed after visualization with or without triamcinolone acetonide, ILM was peeled in a circular motion with the aid of ICG or not. The peeling area was 2 ~ 3 DD in the study by Ikuno [14], 3 ~ 4 DD in the studies by Lam [15], Wei [16], and within the vascular arcade by Li KK [17], Uemoto [18]. After ILM peeling, argon endolaser photocoagulation was performed at the macular hole margin in the study by Lam [15]. Gas tamponade was finally performed using either sulfur hexafluoride (SF6) or perfluoropropane (C3F8) or room air. Patients were instructed to keep prone position for at least 7 days after surgery (Table 2).

**Table 2:** Characteristics of various studies on surgical procedures for myopic macular hole with retinal detachment

Trial, year	Vital dyes	Area of ILM peeling	Type of tamponade gas	Face-down duration	Other treatment	Intraoperative and postoperative complications
Ikuno (2003) [14]	ICG	2–3 DD	14 % or 16 % C3F8	2 weeks	No	Posterior capsule opacification Cataract Recurrent RD
Lam (2006) [15]	ICG	3–4 DD	12 % C3F8/air mixture	7–10 days	Endolaser at the MH margin	Suprachoroidal hemorrhage Iatrogenic retinal breaks Cataract Glaucoma
Li KK (2010) [17]	TA and 0.15 % trypan blue	Between superotemporal and inferotemporal vascular arcades	14 % C3F8	2 weeks	No	Transient increased IOP
Li X (2009) [9]	TA or ICG	NA	14 % C3F8/air mixture	3 weeks	No	Iatrogenic retinal tear Elevated IOP
Nakanishi (2008) [10]	TA and ICG	NA	SF6 or C3F8	At least 1 week	No	Iatrogenic retinal breaks Temporary elevations of IOP Vitreous hemorrhage
Uemoto (2004) [18]	0.25 % ICG	Within the vascular arcade	24 % SF6 or 14 % C3F8 or room air	1 week	No	Iatrogenic retinal breaks Epiretinal membrane Nuclear cataract
Wei (2013) [16]	TA ± 0.5 % ICG ±	3–4 DD	14 % C3F8/air mixture	2 weeks	No	Retinal breaks Epiretinal membrane MH reopen Cataract Glaucoma

*DD* disc diameter, *ICG* indocyanine green, *ILM* internal limiting membrane, *IOP* intraocular pressure, *MH* macular hole, *NA* not available, *TA* triamcinolone acetonide

### Anatomical success

**Fig. 2:**
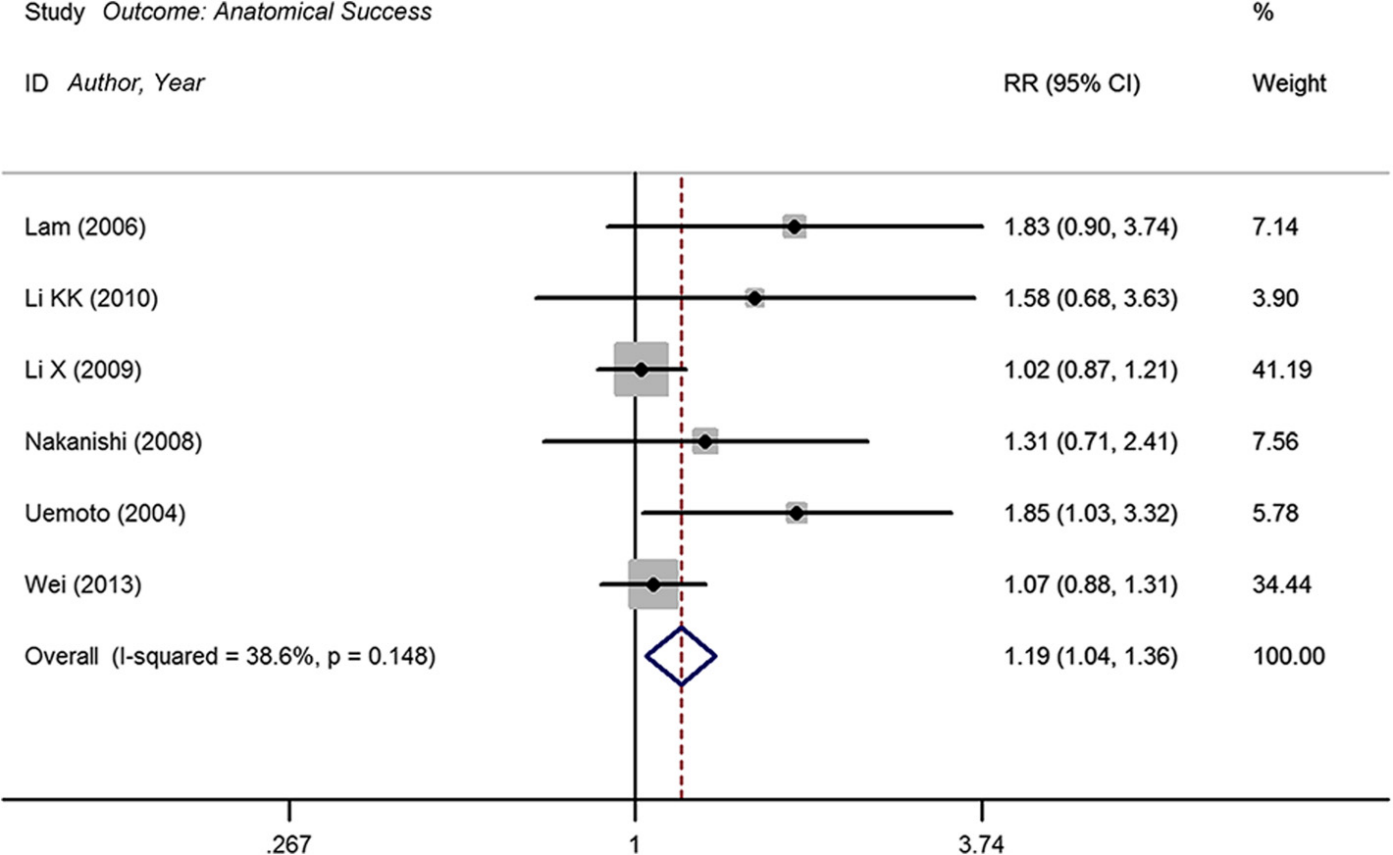
Forest plot of primary anatomical reattachment of internal limiting membrane (ILM) peeling group vs. no peeling group. CI = confidence interval; RR = relative risk

Primary anatomical reattachment was achieved in 173 of 219 patients in the ILM peeling group compared with 96 of 138 patients in the group without ILM peeling. Meta-analysis shows statistically significant RR in terms of primary reattachment between ILM peeling and non-peeling groups (RR, 1.19; 95 % CI, 1.04 to 1.36; *P* = 0.012), with no heterogeneity identified (I^2^ = 38. 6 %; *P* = 0.148) (Fig. 2 and Table 3). Begg’s test (*P* = 0.188) indicated no serious publication bias in these included studies.

**Table 3:** Main results of meta analysis

Outcome	No. of studies	No. of eyes		Test for heterogeneity			Overall effect		
		ILM peeling	ILM preserved	*x* ^2^	I^2^	P	WMD/RR (95 % CI)	Z	P
Primary anatomic reattachment [9, 10, 15–18]	6	219	138	8.15	38.6 %	0.148	1.19(1.04, 1.36)	2.52	0.012
Macular hole closure[14–18]	5	127	83	2.47	0.0 %	0.650	1.71 (1.20, 2.43)	2.95	0.003
Improved BCVA at ≥6 months[14, 18]	2	27	14	0.17	0.0 %	0.677	0.06 (−0.31, 0.44)	0.33	0.738

*BCVA* best corrected visual acuity, *ILM* internal limiting membrane, *RR* relative risk, *WMD* weighted mean difference

### Macular hole closure

**Fig. 3:**
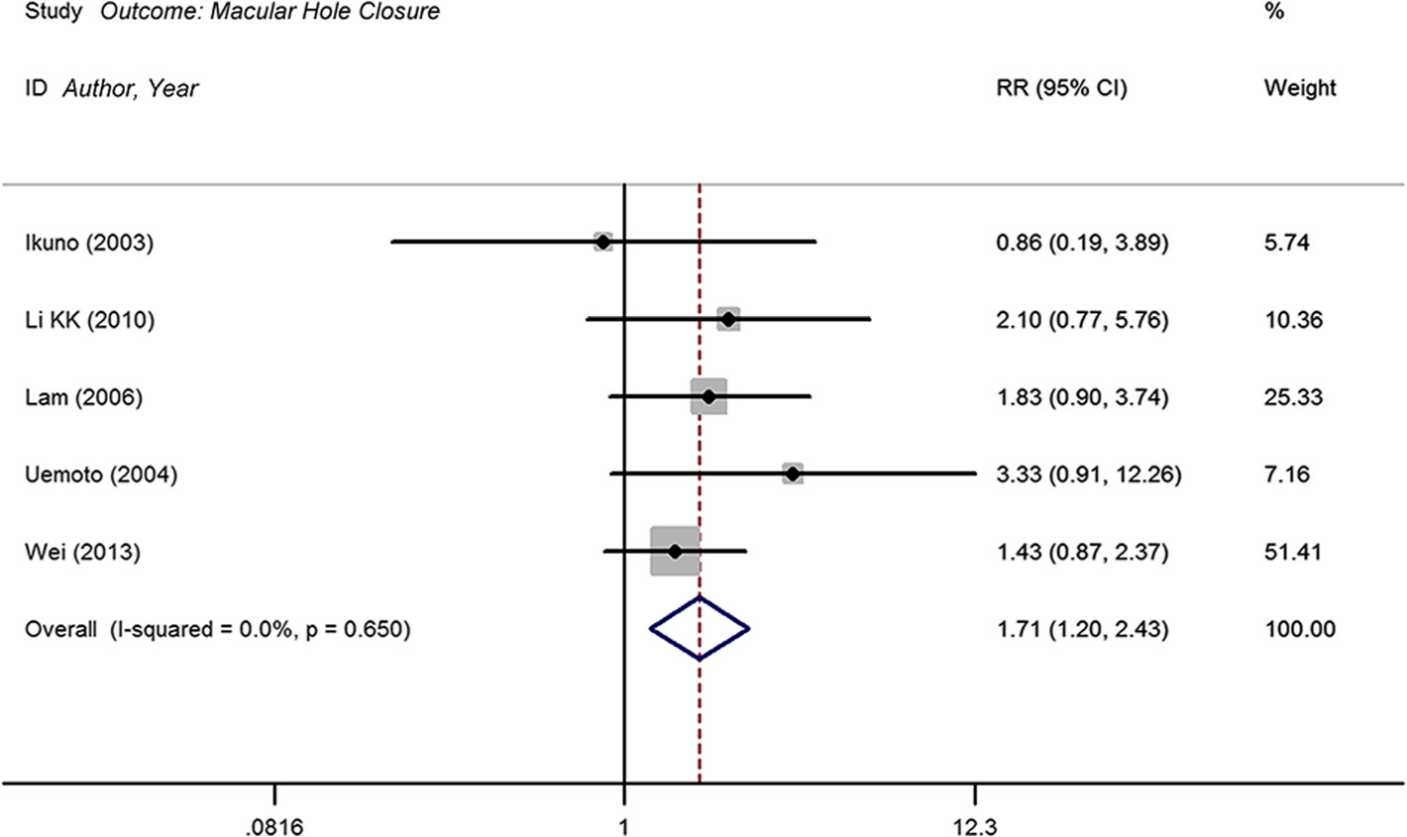
Forest plot of macular hole closure of internal limiting membrane (ILM) peeling group vs. no peeling group. CI = confidence interval; RR = relative risk

Macular hole closure was achieved in 74 of 127 patients in the ILM peeling group compared with 27 of 83 patients in the ILM-preserved group. Meta-analysis shows statistically significant RR in terms of macular hole closure rates between ILM peeling and non-peeling groups (RR, 1.71; 95 % CI, 1.20 to 2.43; *P* = 0.003), with no heterogeneity identified (I^2^ = 0. 0 %; *P* = 0.650) (Fig. 3 and Table 3). The assessment of macular hole closure using Begg’s test (*P* = 0.327) demonstrated no publication bias in included trials.

### Improved logMAR BCVA after surgery at ≥6 months

**Fig. 4:**
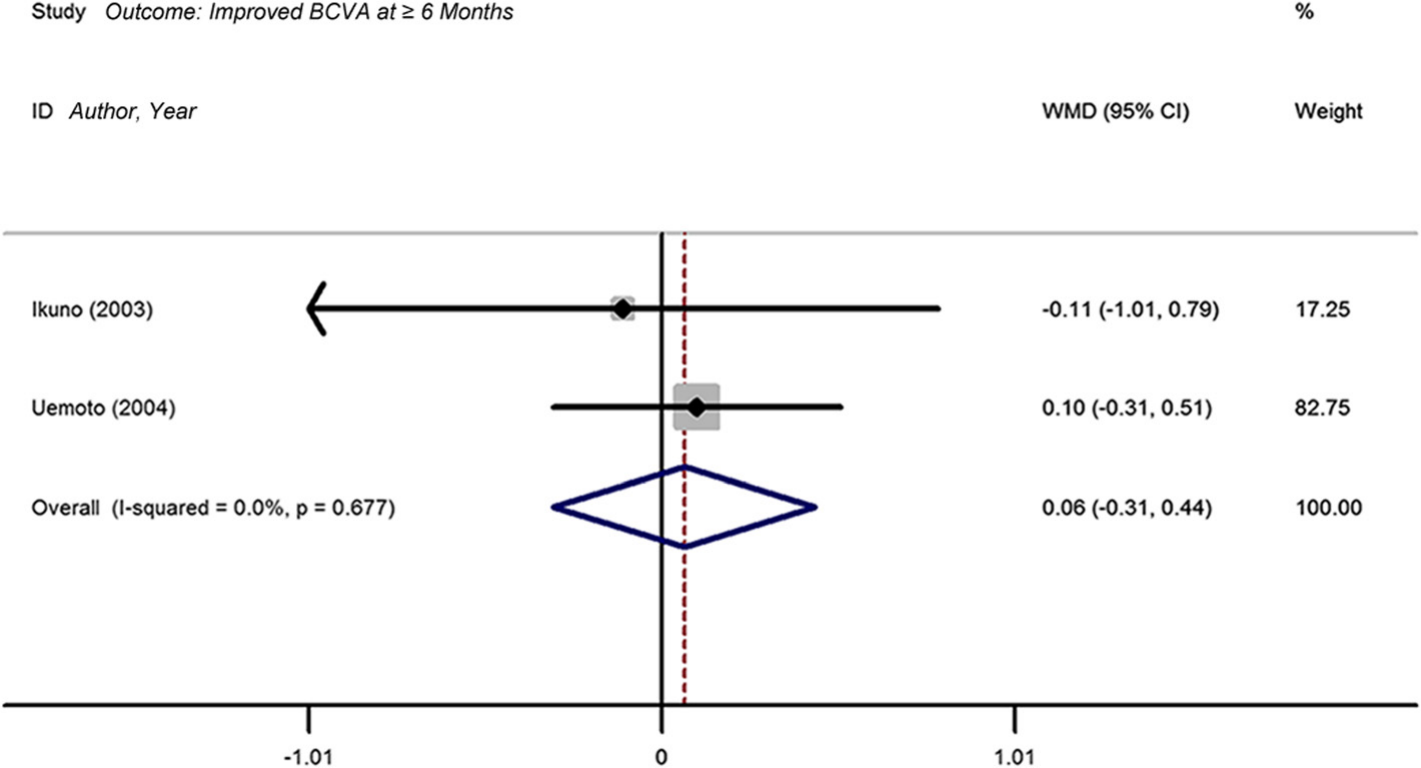
Forest plot of the weighted mean difference (WMD) of improved BCVA at ≥6 months comparing internal limiting membrane (ILM) peeling group with no peeling group. CI = confidence interval

Only 2 studies reported results on improved BCVA for ILM-removed and ILM-preserved groups. The WMD in the improved logMAR BCVA between ILM peeling and non-peeling groups was 0.06 (95 % CI, −0.31 to 0.44). Meta-analysis showed no statistically significant difference (*P* = 0.738), with no heterogeneity identified (I^2^ = 0. 0 %; *P* = 0.677) (Fig. 4 and Table 3). Due to the limited number of trials, publication bias was not assessed.

## Discussion

To the best of our knowledge, this is the first meta-analysis that assesses efficacy and safety of ILM peeling vs. no peeling for myopic MHRD. We reviewed seven comparative studies involving a total of 373 patients who underwent PPV with or without ILM peeling. The pooled outcomes from this meta-analysis, using a fixed effects model, indicated that ILM-peeled group got higher rates of anatomical reattachment success and macular hole closure. However, no significant difference for improvement of BCVA was detected between the two groups with initial surgery. The result from this study provided important findings that may be helpful in the selection of surgical maneuvers.

Six of the trials reported the success rate of the primary anatomical reattachment. Our meta-analysis showed that ILM-peeled group had higher reattachment rate. ILM peeling may help to completely remove macular traction caused by overlying retinal tissues on the ILM and subsequently improve the elasticity of the adjacent retina, aiding in the conformity to the posterior staphyloma [4]. In contrast, in an analysis of the factors predicting anatomical success among patients with myopic MHRD, Lim et al. found that ILM peeling was not a significant predictor of success. They suggested that the role of ILM peeling for tangential traction relief was diminished by the elongation of axial length [8].

Studies included in our meta-analysis showed that macular hole closure rate in ILM peeling group ranged from 42.9 to 70.5 % (results not shown). Statistical difference was identified between ILM peeling group and non-peeling group. In a recently published meta-analysis evaluating the effects of ILM peeling for full thickness macular hole, Spiteri Cornish et al. found evidences favoring ILM peeling in terms of primary and final macular hole closure [19]. ILM may act as a scaffold for cell migration and consequent fibroglial proliferation. Thus, ILM peeling was considered to relieve the contraction of epiretinal cellular constituents adjacent to the macular hole. In addition, the tangential traction of residual vitreous or epiretinal membranes (ERMs) may be completely released after ILM removal. These factors can ensure better closure of macular hole and recovery of macular shape [20].

Our study indicated that no obvious visual improvement was achieved in these myopic eyes with MHRD, in despite of anatomical and surgical success. Similarly, in a study to evaluate the efficacy of PPV for each stage of myopic macular traction, no difference of postoperative BCVA was found between patients with and without ILM peeling [21]. Besides anatomical success and macular hole closure, visual acuity can be affected by many other factors that are hard to assess. The duration of the detachment, the time of surgery and the previous changes, including atrophy of the RPE and choriocapillaris at the posterior pole, may be influential factors for the final visual outcomes in myopic MHRD. Additionally, it could take longer time to observe the effect of ILM peeling on the visual acuity, but the studies with a longer follow-up are largely missing.

ILM peeling has been considered to cause mechanical retinal damage, including physiological alterations in Müller cells [22], irregularities of nerve fiber layer [23], small paracentral scotomas [24], loss of Müller cell endfeet within the peeling area and weakening of macular glial structure [25, 26]. ILM removal even resulted in the development of postoperative full-thickness macular holes in some cases of myopic foveoschisis [27]. More recently, a retinal dimple sign was identified in highly myopic eyes after ILM peeling by an image of en face OCT [28]. Additionally, due to the osmolarity effects or phototoxic properties, studies showed that ICG may have potential retinal toxicity with injury to RPE cells and ganglion cells during ILM peeling [29, 30].

There are growing evidences favored the effects of ILM peeling for MHRD. It was reported that fibroblast-like cells, collagen fibrils and acellular collagen fibers have been detected by transmission electron microscopy on the vitreous surface of ILM in myopic MHRD [31]. ILM peeling was believed to remove the collagen fiber and cellular constituents that may be responsible for gliosis stimulation and foveal traction in myopic maculopathy, indicating the efficacy of ILM removal for MHRD. In addition, ILM peeling was considered to be one of the important prognostic factors associated with a higher anatomical success rate for retinal detachment resulting from myopic macular hole [15]. However, due to the retinal thinning and friability in severely myopic patients, ILM peeling is technically more difficult, even assisted with effective dyes and performed by experienced vitreoretinal surgeons [11]. In the pathologic myopia, axial length elongation and posterior staphyloma formation often result in retinal thinning at macula area, peeling of ILM is likely to pose an increased risk for surgical trauma. Future studies are needed to make clear the safety of ILM peeling for retinal detachment arising from macular hole in highly myopic eyes.

It is worthy to note the relatively limited powers of our meta-analysis when considering the results. First, this study was limited by the low quality of the retrospective studies included and the lack of randomized controlled trials-based evidences. Most studies were carried out with small sample size, nonrandomized nature or no double blinding. The only randomized study did not show any outcome of macular hole closure, whereas other studies were more likely to be prone to publication bias. Although the study quality was assessed, the system may not assign sufficient importance to the comparability of the data from the peeling and non-peeling groups. This may compromise the efficacy of this instrument in controlling potential biases. In addition, results on improved BCVA were only obtained from 2 studies, which may restrict its reliability. Moreover, the follow-up duration was different in each included study, which may also result in difference and corresponding biases. Thus, the interpretation of the results may be greatly affected. Second, this study was limited to use published index, therefore the papers, particularly those published in languages other than English may have failed to be included. Third, the parameters of interest may have a large degree of heterogeneity. For example, due to the potential complications of long-term use, silicone oil was generally reserved for redetachment cases in most studies. In this analysis, we excluded the studies with silicone oil tamponade in primary surgery. Last, adjunctive procedures were dependent and postoperative management was variable among each surgeon, which may affect the final outcomes.

However, in this study we had the detailed protocol before initiating the analysis, the explicit methods for trials selection, and data extraction, which helped to minimize the likelihood of bias.

## Conclusions

This study suggests that ILM peeling is a more effective and safer procedure in highly myopic eyes with MHRD, but it does not seem to offer significant visual advantages compared with non-ILM peeling. More standard-designed studies with prospective randomized control, incorporating larger sample sizes, and long-term follow-up could help to provide more reliable evidences.

## Abbreviations

BCVA, best corrected visual acuity; CI, confidence interval; ILM, internal limiting membrane; logMAR, the logarithm of the minimum angle of resolution; MHRD, macular hole retinal detachment; PPV, par plana vitrectomy; PRISM, preferred reporting items for systematic reviews and meta-analyses; RR, relative risk; WMD, weighted mean difference
